# Translating E-Mental Health Into Practice: What Are the Barriers and Enablers to E-Mental Health Implementation by Aboriginal and Torres Strait Islander Health Professionals?

**DOI:** 10.2196/jmir.6269

**Published:** 2017-01-11

**Authors:** James Bennett-Levy, Judy Singer, Simon DuBois, Kelly Hyde

**Affiliations:** ^1^ University Centre for Rural Health University of Sydney Lismore Australia

**Keywords:** e-mental health, indigenous populations, Aboriginal and Torres Strait Islander peoples, professional supervision, professional consultation, service implementation, health education, mobile apps

## Abstract

**Background:**

With increasing evidence for the effectiveness of e-mental health interventions for enhancing mental health and well-being, a growing challenge is how to translate promising research findings into service delivery contexts. A 2012 e-mental health initiative by the Australian Federal Government (eMHPrac) has sought to address the issue through several strategies, one of which has been to train different health professional workforces in e-mental health (e-MH).

**Objective:**

The aim of the study was to report on the barriers and enablers of e-MH uptake in a cohort of predominantly Aboriginal and Torres Strait Islander health professionals (21 Indigenous, 5 non-Indigenous) who occupied mainly support or case management roles within their organizations.

**Methods:**

A 3- or 2-day e-MH training program was followed by up to 5 consultation sessions (mean 2.4 sessions) provided by the 2 trainers. The trainer-consultants provided written reports on each of the 30 consultation sessions for 7 consultation groups. They were also interviewed as part of the study. The written reports and interview data were thematically analyzed by 2 members of the research team.

**Results:**

Uptake of e-MH among the consultation group was moderate (22%-30% of participants). There were significant organizational barriers to uptake resulting from procedural and administrative problems, demanding workloads, prohibitive policies, and a lack of fit between the organizational culture and the introduction of new technologies. Personal barriers included participant beliefs about the applicability of e-MH to certain populations, and workers’ lack of confidence and skills. However, enthusiastic managers and tech-savvy champions could provide a counter-balance as organizational enablers of e-MH; and the consultation sessions themselves appear to have enhanced skills and confidence, shifted attitudes to new technologies, and seeded a perception that e-MH could be a valuable health education resource.

**Conclusions:**

A conclusion from the program was that it was important to match e-MH training and resources to work roles. In the latter stages of the consultation sessions, the Aboriginal and Torres Strait Islander health professionals responded very positively to YouTube video clips and apps with a health education dimension. Therapy-oriented apps and programs may fit less well within the scope of practice of some workforces, including this one. We suggest that researchers broaden their focus and definitions of e-MH and give rather more weight to e-MH’s health education possibilities. Developing criteria for evaluating apps and YouTube videos may empower a rather greater section of health workforce to use e-MH with their clients.

## Introduction

### A National E-Mental Health Strategy

e-Mental Health (e-MH) is gaining traction, nationally and internationally, as a way to increase access to mental health services and deliver treatment in a cost-effective manner [1]. Research suggests that for some common mental health problems, e-MH treatments can be just as effective as face-to-face treatments [2], although in service delivery contexts, considerable variability is reported [3,4].

Drawing on the reported positive impacts of e-MH and the potential to increase access to mental health services for all Australians [5], the Australian Federal government developed a national e-MH strategy [6]. One element of this was the development of a national Web-based therapy service, the MindSpot Clinic [4,7]. Another element was the e-Mental Health in Practice (eMHPrac) project, an initiative to provide health professional training in both patient-facing and provider-facing e-MH interventions.

### E-Mental Health in Practice

The Australian government established eMHPrac initially as a 3-year project (2013-16), recognizing that, in order to translate e-MH from research to clinical practice, health provider training was a prime requirement. Since the e-MH role of health providers (eg, general practitioners vs psychologists) tends to differ (eg, appropriate referral vs providing guided self-help), separate arms of the project were established to train 3 groups of health providers: GPs, allied health professionals, and health professionals providing services to Aboriginal and Torres Strait Islander peoples. This study focused on the experiences of a group of Aboriginal and Torres Strait Islander health professionals who were providing services to Aboriginal and Torres Strait Islander peoples.

### Aboriginal Mental Health

The extent of mental ill health and psychological distress in Aboriginal and Torres Strait Islander communities has been well documented and closely linked to intergenerational trauma and to current levels of disadvantage and disengagement [8]. Aboriginal and Torres Strait Islander adults are almost 3 times more likely to experience high or very high levels of psychological distress than other Australians; have high rates of substance abuse; and have 2-5 times the rate of suicide compared with other Australians [9-11]. Indigenous citizens of other westernized countries (eg, Native Americans, Inuit, Maori) also experience high degrees of psychological distress and historical trauma [12-14], which suggests the possible relevance of our project for other Indigenous peoples.

### Availability and Potential of e-MH for Indigenous Peoples

e-MH research with Indigenous peoples has been hampered by a lack of culturally appropriate e-MH resources. At the start of our project, there were already many Web-based therapy programs and apps available for majority cultures and a large body of outcome studies. In contrast, there have still not been, to our knowledge, any e-MH therapy outcome studies for Indigenous peoples from any country.

When we started the study, the availability of e-MH therapy resources for Australia’s Indigenous peoples was restricted to just one Indigenous-specific app, Stay Strong, which could be downloaded onto iPads. Stay Strong is a strength-based app with specific Indigenous content and imagery [15], which has been designed for health workers to use with Indigenous clients. For a Youtube video demonstration of Stay Strong, see [16]. Stay Strong uses cognitive behavioral principles to (1) set SMART goals, (2) build social support, and (3) identify and change behaviors that are either enhancing or diminishing mental health. An earlier paper-and-pencil version of Stay Strong had proved to be valuable in working with Aboriginal and Torres Strait Islander clients [15]. The iPad version had previously received strong endorsement from our local advisory groups and learning circles and from Northern Territory health professionals and community members as acceptable and appropriate [17,18]. Hence, Stay Strong was included as a core part of the training program.

### Provision of Training in Culturally Appropriate e-MH for Aboriginal and Torres Strait Islanders

With regard to culturally appropriate e-MH training, there has been an initial eMHPrac report from a Northern Territory training program showing that practitioners reported gains in knowledge, skills, and confidence in use of Stay Strong when comparing pre- and immediate posttraining measures [19]. The study was consistent with other reports in the literature showing that one-off workshops can influence practitioner knowledge and attitudes [20]. However, to date, no study has addressed day-to-day e-MH implementation with Indigenous peoples in Australia or elsewhere.

Several other studies have suggested that “low-intensity interventions” delivered by health providers without specialist knowledge of psychological therapies may potentially be useful, if delivered in a culturally appropriate way [15,21-23]. Hence, the current training included Aboriginal health providers with limited knowledge of psychological therapies.

### The Importance of Follow-Up Supervision or Consultation Sessions

A burgeoning literature on the impact of one-off workshops now suggests that training without follow-up supervision, consultation, or reflection is largely ineffective in influencing therapist behavior and patient outcomes [24-26]. Therefore, for best results, it appears that follow-up consultation or supervision should be offered if practitioners are to gain maximum benefit from training programs.

Accordingly, for our study, Aboriginal and Torres Strait Islander health providers who received a 2- or 3-day training in e-MH strategies (the R U Appy program) were offered monthly follow-up consultation sessions to reinforce learning from the training program. The distinction between consultation and clinical supervision is that consultation focuses on the further development of specific intervention techniques, and is usually provided by experts who are external to an organization; while clinical supervision usually focuses on the practitioner’s clinical work with specific patients, and is often provided by a senior staff member internal to the organization [25].

A dual consultation model with one Aboriginal and one non-Aboriginal consultant-trainer was developed for our project to provide both e-MH expertise and cultural safety. This was based on a proposal from a recent study of issues affecting supervision and support for the Aboriginal and Torres Strait Islander mental health workforce [27].

### Purpose of the Study

Ultimately, the goal of training health professionals in e-MH is to facilitate the uptake of e-MH amongst Aboriginal and Torres Strait Islander peoples. As a first step, the aim of our study was to provide a qualitative evaluation of the impact of e-MH training plus follow-up consultation sessions with Aboriginal health providers. To our knowledge, this is the first study, in Australia or elsewhere, which has provided extended e-MH training/consultation to Indigenous health professionals, and then examined the enablers and barriers of implementation.

## Methods

### Intervention: Training Plus Follow-Up Consultation Sessions

#### Training

A total of 50 providers of health and community services for Aboriginal and Torres Strait Islander peoples attended either a 3-day (n=43) or a 2-day (n=7) R U Appy e-MH training in one of the 2 locations (Lismore or Tweed Heads) in northern New South Wales. The aim of the R U Appy training program was to improve awareness, confidence, and competence in the use of e-MH resources. The design of the program (workshops plus consultation sessions) was guided by the extensive involvement of local advisory groups, learning circles, and a regional Aboriginal and Torres Strait Islander Health Council [28]. R U Appy training was strongly oriented toward experiential learning, featuring a hands-on approach for using iPads to search the Internet and download and use apps. One of the 3 training days was specifically devoted to training health providers in the Aboriginal-adapted app, Stay Strong. One participant had used the paper-based version of Stay Strong, but none had previously used the app.

#### Follow-Up Consultation Sessions

At the completion of the R U Appy workshop program, the trainees were invited to join a skills-based consultation group. Those wishing to attend were assigned a group based on either geographic location or organization-specific groupings. For example, where there were several participants from the one organization, an organization-specific group was formed (4 groups), and the sessions were held in the premises of the organization. Alternatively, participants from different organizations working in the same region formed “location-specific” groups (3 groups) and agreed on a specific meeting place.

Participants and managers were provided with the rationale for the consultation sessions and encouraged by the R U Appy trainers to attend following the training. Some participants were actively supported by their managers to attend both workshops and consultations; other managers viewed the consultation sessions as optional for their staff. Equally, some workers were very keen to participate in consultation sessions; others, for various reasons, had little interest or capacity to attend (further exploration of these issues is the subject of a parallel study currently underway).

The consultation sessions were formulated as a skills-based follow-up to the R U Appy training, drawing on research indicating the importance of consultation or supervision for embedding new skills [29,30]. They were conducted monthly for each group by the 2 e-MH trainers. One was a male psychologist specialized in e-MH, and the other a female Aboriginal Mental Health Worker undertaking an undergraduate degree in psychology. The initial consultation sessions were primarily focused on embedding Stay Strong skills; later sessions were adapted to meet other needs of participants (see Results).

#### Data Collection

The study data were the consultant-trainers’ (SD, KH) written reports on each of the 30 consultation sessions that they conducted. Immediately following each supervision session, a report was written by SD and then checked by KH. The consultant-trainers were asked to record: who was present, whether they had appropriate technology, and how many participants had used Stay Strong or another e-MH resource with clients. The session reports also contained details of session activities, session observations, and participant reports of experiences that had facilitated or hindered their learning. These reports were then emailed to the research team (JS and JBL) that reviewed the content and systematically stored the reports (see Multimedia Appendix 1 for an example of a consultant-trainer session report). The consultation sessions were loosely structured to enable the participants to direct the sessions according to their specific needs. As such, the consultants’ reports did not follow a structured reporting schedule; rather they described the different or divergent group experiences.

At the completion of the consultation sessions, the research team conducted a semistructured interview with the consultant-trainers (see Multimedia Appendix 2 for the interview guide). The purpose of the interview was to allow more detailed exploration of key issues emerging from their reports. The primary focus of the interview was on consultant observations about participants’ interest, confidence, and use of e-MH resources and the factors that impeded or facilitated their learning. The interview was digitally recorded and transcribed verbatim for data analysis (see next section).

#### Data Analysis

At the end of the 6-month consultation period, the session reports were collated in date order for each of the 7 consultation groups and compiled into 1 document. One member of the research team (JS) conducted the initial thematic analysis of the session reports, following best practice guidelines to explicate “motivations, experience, and meaning” [31]. The process involved reading the 30 field note reports several times to develop broad categories, and then rereading the dataset and assigning code names to the emerging themes. Subsets of the data were then read again to check the relevance of the code names.

In order to enhance rigor and validity, a second researcher (JBL) randomly selected 6 of the field note reports and repeated the process. Comparison of the 2 categorizations revealed consistency across categories and agreement on codes and key themes. Once categorization of the reports was completed, a similar process was then undertaken with the interview transcript.

**Figure 1:**
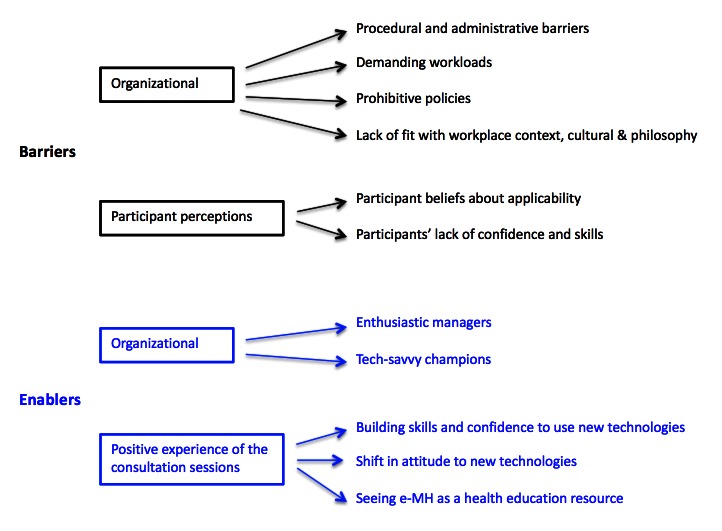
Barriers and enablers to the uptake of e-mental health practices by Aboriginal and Torres Strait Islander health professionals.

A review of both datasets (reports and interview transcript) indicated that all the themes identified in the reports were represented in the analysis of the interview transcript. Further themes were identified in the analysis of the interview, as this dataset contained a more detailed and nuanced description of the consultation process. A combined thematic map was developed linking the two datasets (see Figure 1).

As a further check on the validity of the analysis, the thematic map, the quotations and examples, and the interpretation of the data were reviewed by the consultant-trainers. This review indicated that the data and themes were found by both consultant-trainers to be wholly consistent with their experience.

In the Results section:

1. The quotes in italics are those of the consultant-trainers.

2. Double quotation marks are used to represent the health professionals’ “voice” as quoted by the consultant-trainers.

3. CTR refers to quotes taken from the consultant-trainers’ reports.

4. CT1 and CT2 refer to quotes from the interviews with them.

The study received ethics approval from the Aboriginal Health and Medical Research Council (NSW) AHMRC-HREC 955/13 and the Northern NSW Local Health District NCNSW-HREC 076.

## Results

### Participants

Twenty-eight of the 50 health providers, who attended the 2- or 3-day R U Appy training program in northern New South Wales, elected to attend the consultation sessions. Twenty-two R U Appy participants were not able to attend consultation sessions or chose not to. Two of 28 consultation study attendees were excluded from our analysis as they were not seeing clients at the time of the consultation sessions. Of the 26 health professionals in our study, 14 were females and 12 males. Twenty-one were Aboriginal and Torres Strait Islanders, and 5 were from non-Aboriginal backgrounds. Participants were drawn from 10 organizations: Aboriginal-specific nongovernmental organizations (NGOs) (3), generalist NGOs (6), and government health services (1). The mean consultation group size was 4.0 people (range 2-7). The mean number of consultation sessions attended was 2.4 sessions (range 1-5).

Participants had a variety of job titles, mainly describing support or case management roles (eg, youth worker, indigenous services worker, drug and alcohol worker, family development worker, well-being coordinator, Aboriginal health education officer, mental health support worker, and healthy lifestyle worker). Most had obtained certificates or diplomas from industry training programs (eg, Certificate in Primary Health Care); 2 had undergraduate Bachelor degrees, one of whom also had a Master degree. None of the participants had qualifications in a mainstream health profession (eg, nurse, psychologist, social worker), or specialist training in an evidence-based psychological therapy.

### Usage of e-MH Resources

The study protocol and initial consultation sessions were focused on building Stay Strong skills. However, after 2-3 sessions, it became apparent to the consultant-trainers that some participants were not able to gain regular access to iPads with Stay Strong; other participants noted that Stay Strong did not fit with their work contexts, and wanted to broaden the focus of consultation sessions to a wider range of well-being resources (not specifically “mental health”) such as specific Aboriginal health education resources, YouTube videos, and a broader range of apps. These observations led the R U Appy team to adapt the protocol to better meet the needs of participants, with consultation content becoming largely participant-determined. This change in emphasis was a key factor in maintaining group engagement.

The consultants’ reports illustrated that 23% (6/26) had used Stay Strong with 1 client (or more) over the period of the consultation sessions; only 1 participant was using Stay Strong regularly with clients. Also, 31% (8/26) reported using other e-MH resources (YouTube, other apps) between consultation sessions.

### Enablers and Barriers to the Uptake of e-MH

The main themes that emerged from the analysis of the consultation notes and interview were clearly identifiable as (1) the barriers and (2) the enablers to the uptake of e-MH. Figure 1 summarizes the barriers and enablers.

### Barrier 1: Organizational

The consultant-trainers noted that the participants were hampered in various ways by organizational issues and structures that prevented the uptake of e-MH. These organizational barriers included procedural and administrative obstacles, demanding workloads, and entrenched and obstructive IT policies within the workplace.

#### Procedural and Administrative Barriers

In some services, the lack of procedures to manage iPad security and storage blocked accessibility to the devices:

We had some organizations where we would go to a consultation session and a person would say, “I still haven’t loaded (the Apps) onto the iPad and we can’t quite get to them because they are locked in a bottom draw”

CT1

In other organizations, the management of the iPads was the responsibility of senior staff who were often too busy to address the IT requirements:

(The worker) has been knocking on the manager’s door to progress the IT side, which is to get (the app) downloaded onto the iPad, but (the worker) is getting drowned out by the other more immediate needs of the organization

CT1

The consultants described these issues by stating that the workers were often “caught up in an administrative mousetrap” [CT1].

In some situations, it was the dearth of basic procedures to guide the management of new technologies that stymied implementation:

(The worker said) “I’ve got the iPad and I’m keen to download (the App), but the person who bought the iPad and set it up left the organization and we don’t know the code to get into the iPad...the critical piece of information has gone with someone else”...there can be real enthusiasm, but the challenges of technology can jump up quite quickly

CT1

The consultants reported numerous technological hurdles experienced by the health professionals as they attempted to engage with new technologies and specifically with iPads. Many of these obstacles stemmed from a lack of technological capability within the organization. For instance, managers or team leaders with responsibility for the iPads may not be tech savvy themselves:

We are also asking (managers who have not attended training) if they know how to use an iPad...how to turn it on, do they have an iTunes account … (some people think) you just turn it on and it’s there

CT2

#### Demanding Workloads

The consultants reported that, in many services, demanding workloads left the workers with little or no opportunity to incorporate new skills into their existing work practices:

Another stumbling block is the sheer busy-ness that people have in their work lives...they are too busy to include (e-MH)

CT1

Consultants also commented that the high turnover of staff in some organizations was problematic:

As an organization, they're certainly taking it on, I think the fly in the ointment was just that...we lost maybe half or three quarters of the staff that went through the training…so there was a knowledge/experience leak from the training

CT1

#### Prohibitive Policies

The consultants also reported that several health professionals worked in government-funded services where the organizational policies prohibited purchase and use of new technologies:

Their workplace don’t have iPads so using e-technologies is made difficult … (they were) frustrated with being sent to training but then not being able to apply the tools back at work

CTR

(The workers said):

“it’s a great app, but we're not going to be able to do it because management's just not going to give us the resources to do it”

CTR

#### Lack of Fit With Workplace Context, Culture, and Philosophy

Some workers perceived a “lack of fit” between e-MH resources and their current work practice. For instance, Stay Strong, which was valued and used by some workers, did not fit with the work contexts of others:

(Some workers) don’t feel that the nature of their contact with clients, which is usually crisis driven and concerned with meeting immediate living needs, gives the opportunity to use the app (Stay Strong)

CTR

(The workers reported that they are) dealing with very fundamental needs as an organization...finding housing, getting their forms sorted out, dealing with them being safe (and) keeping peoples’ heads above water, practically

CT1

The culture and philosophy of the organization also impacted on the uptake of e-MH resources. For some workers, technology was perceived to reduce human connection:

(These workers) principally value face-to-face contact with clients and the “human connection”...they want to “connect with the person” and assist them to connect emotionally to those around them. Technology was still viewed as an impediment to this

CTR

(Another worker reported to the consultants that) “it’s hard to engage with our clients at the best of times, let alone trying to show them a website to use”

CTR

### Barrier 2: Participant Perceptions

In addition to organizational barriers, the consultants reported that the workers’ perceptions about e-MH resources also posed barriers to their uptake.

#### Participant Beliefs About Applicability

Another view reported by the consultants related to generational beliefs about technology:

(Workers said) it’s more for the younger generation, and we're dealing mainly with workers that were in the older section

CT2

(Some workers said) older clients feel uncomfortable with technology as it’s not part of their everyday experience

CTR

#### Participants’ Lack of Confidence and Skills

The consultants noted that, for some health professionals, a perceived lack of confidence and skills using new technologies hindered uptake:

The workers that weren't feeling 100% confident using iPads would say, “if I'm clumsy, then that takes away that rapport with the client”

CT2

What I found (was) if they didn't feel confident with using the app they refused to use it within clients...If they couldn't do it within themselves, why should they have to get a client to do it?

CT2

Collectively, these issues highlighted the complexity involved in the implementation of e-MH, particularly for organizations that had limited resources and limited expertise with new technologies.

### Enabler 1: Organizational

#### Enthusiastic Managers

The consultants reported how the influence of enthusiastic managers significantly enhanced the workers’ interest and opportunity to engage with e-MH resources. For instance, the manager of one service attended the 3-day R U Appy workshops and participated in the consultation sessions:

(Although) he had limited experience with technology, he identified the importance and value of his organization staying abreast of these technologies so as to remain in touch with younger clients and the organization as a whole growing into a more technologically-driven world

CTR

The consultants also reported that a number of managers were supportive and had purchased iPads specifically to enable staff to develop e-MH skills and competency:

It helped having the service (manager) on board...some services had purchased iPads and…were keen to be using more e-technologies

CT2

#### Tech-Savvy Champions

One organization had a “tech-savvy champion” who enthusiastically embraced e-MH resources and inspired others to do so:

He acted as a champion in his organization and was encouraging other staff members to get involved … one idea he had was having a staff-client day that was specifically orientated towards practicing with the Stay Strong app

CT1

### Enabler 2: Positive Experience of the Consultation Sessions

#### Building Skills and Confidence to Use New Technologies

Many workers began the R U Appy training with little or no experience of using new technologies. Through the consultation process, the consultants described how some workers surprised themselves by developing skills in using new technologies and moved from being “dinosaurs” to “e-explorers”:

She was very adamant (stating) “I’m a dinosaur, and I can’t use these things” … she’s gone from (being) a dinosaur to “oh my god, I’ve learned how to do this”

CT2

(Another worker) was like, “oh my God, I’m a dinosaur,” and at the end of the training, low and behold, he went and bought himself an iPad, and sits on Facebook and is willing to do the Stay Strong app. He was another one that had done a big turnaround

CT2

#### Shift in Attitude to New Technologies

Associated with the development of new skills, the consultants also described how some workers changed their views from “technology is bad” to “technologies can be helpful”:

People would come with quite strong ideas around technology, (they) can see a lot of detriments associated with technology use...bullying online, young people being exposed to information and things through their devices that can be quite harmful. For a lot of health workers, it's an intrusion on society that's been quite damaging. I think it was quite enlightening (for them) to see that there had been a lot of resources developed that are aimed to assist and support people and that they can take advantage of this technology (for the) people they work with

CT1

They didn’t realize how many resources are actually out there. So they’ve gone from hardly knowing anything, to “wow,” (all the) YouTube clips and different apps and websites

CT2

The consultants also reported having “complex conversations” with workers during the consultation sessions “(about) how (technology) fits with their work environment, how it fits with how they engage with people” [CT1]. They described one particular worker’s experience during the consultation process in shifting his understanding about the potential relevance of new technologies in his work with younger clients:

(A worker) and I were having a conversation about how the youth have a good way of emotional expression on text, rather than face-to-face conversations. As we were having that conversation, (another worker) started thinking of (a young client) that he could actually use technology with, and get him to build a rapport with this young one, because he (the young client) was so technologically savvy

CT2

The consultants noted the various views expressed by the health professionals about their motivation to become tech-savvy. For some, it was the view that

“technology is becoming part of everyday life.”

CTR

For others, engaging with technology was important in order to

“guide and educate people in the healthy use of technologies”

CTR

and as workers, the importance

“to participate with technology in order to not be left behind”

CTR

#### Seeing e-MH as a Health Education Resource

Responding to participants’ expressed needs for apps for different purposes and contexts, the consultants introduced a range of different e-MH resources into the consultation sessions, tailoring them to the specific interests of the different consultation groups. A feature of the consultation sessions was the opportunity for workers to learn how to search for and assess resources that were relevant to their work context:

(It’s been about) becoming aware of the resources that are out there...that was certainly a big impact...thinking a bit more critically about the role that technology plays in the lives of their clients, and then how they may be able to interface with that in a way that actually supports them in the work that they're doing with clients

CT1

Many of the health professionals worked in the drug and alcohol field and found some of the new technologies to potentially offer a valuable adjunctive educational tool:

Having something else to explain what an addiction looks like and then having a library of YouTube clips that you could refer to and have a different way of engaging…it’s a different way of having a discussion about that issue

CT1

We showed them some of the YouTube clips of what an addiction can look like. (Workers) took that on board and could see how they could use that within their service...how the brain works, if you’re taking a drug what it can do to a brain

CT2

YouTube, as a medium, was seen as particularly valuable for Aboriginal and Torres Strait Islander clients. One of the consultants remarked:

Aboriginal people, (are) so visual, that having those YouTube clips we can actually connect to that visual stuff, and go, oh, that’s exactly what you're talking about, we know that feeling

CT2

## Discussion

### Principal Findings

As research evidence for the value of e-MH resources accumulates, attention is gradually turning to strategies for e-MH implementation in health service contexts [4,32,33]. For our study, we designed a training plus consultation program to teach e-MH skills to Aboriginal and Torres Strait Islander health and community professionals, drawing on best practice implementation science [25,34]. A previous study had identified the potential value of culturally appropriate consultation or supervision sessions to follow up training for Aboriginal health providers [27]. However, our study is the first to follow up Aboriginal and Torres Strait Islander health providers over 6 months, and examine the enablers and barriers to e-MH implementation. Furthermore, no reports of training plus follow-up consultation sessions with Indigenous health workers from other countries are available.

Among those who attended consultation sessions, interest in e-MH was strong and the consultation sessions were well received and appreciated. Stay Strong, the only Indigenous-specific app available at the time of the training, was well accepted by our participants, mirroring reports from Northern Territory community members [18], but it was only used by a minority of workers (6/26) even after training plus consultation. However, interest in other e-MH resources grew as the sessions developed. In particular, Aboriginal and Torres Strait Islander workers expressed interest in apps and YouTube videos, which could be used for health education and information purposes. Eight out of 26 workers started to use apps and e-MH resources other than Stay Strong with clients.

While the translation-to-practice impact of the training and consultation might be perceived as only moderate, qualitative session-by-session analysis of the consultation sessions reveals that there were good reasons why uptake was relatively low. Organizational impediments played a major role in determining whether participants adopted e-MH practices, as has been noted in e-MH studies with non-Indigenous health providers [35]. Our participants reported difficulties in accessing iPads, passwords, and iTunes accounts; pressing needs of other work and relegation of e-MH to low priority; and high staff turnover. Organizational culture, orientation, and individual factors also had an impact [35,36]: one organization and its staff saw technology as a barrier to human connection; others were largely responding to crises and contexts where e-MH might have been inappropriate, or viewed e-MH as valuable only for young people. The perception of “fit” with the work environment, work roles, clients, and the culture of the organization was central to workers’ willingness to adopt e-MH [37]. However, organizations could exert a positive impact too. Managers who supported e-MH and tech-savvy champions in the organization tended to influence other workers in their organization and provide enhanced access to e-MH resources.

The qualitative analysis indicated that the consultation sessions had a positive influence in a number of ways. They enhanced curiosity and interest in e-MH, facilitated exploration, increased skills in some participants, and created major shifts in attitude toward e-MH. Despite this, some workers simply did not feel confident enough in their skills to use e-MH resources and thought that their clumsiness might affect rapport with clients.

### Conceptualizing the Results

Perhaps the most interesting impact of the consultation sessions was the change of tack taken by the consultants around the third session when they realized that, for some organizations and health providers, there was a lack of fit between the Stay Strong app and workers’ roles and organizational cultures. To grasp this point, it is important to understand that only 2 of the participants had university-level training, and none were in a specialized mental health profession (eg, psychologist, social worker, or physician). For most Aboriginal and Torres Strait Islander health providers in our region, their scope of practice was restricted to an educational role or a support worker or case management role rather than a therapy role.

Once the consultant-trainers recognized that Stay Strong lay outside of many participants’ scope of practice, they found that participants’ interest was piqued by the possibility of using apps and YouTube clips for health education and information purposes (see Multimedia Appendix 3 for examples). The health providers were genuinely excited at this prospect as there is high usage of mobile phones in the Aboriginal and Torres Strait Islander population [38], and a perception that their clients are often very “visual” (see last comment in Results section).

Reynolds et al have recently developed conceptual model to understand the value of different e-MH resources for different types of health worker [37]. The model helps to shed light on why Stay Strong may have been favored by some workers but not by others, and why educational YouTube clips and apps were strongly favored by this cohort. The model makes important distinctions between the different roles of workers (eg, nonclinical, case management, coaching, therapist) and the relative value of different e-MH resources to them. An implication of the model (p. 7) is that Stay Strong may be most suited to workers who have mental health skills appropriate for coaching or therapist roles. However, the majority of the Aboriginal and Torres Strait Islander health workers in our cohort had more of a support or health education role than a coaching or therapist role. What the Reynolds et al model suggests is that the most appropriate e-MH role for this workforce is to provide e-MH information and health education resources for their clients, which is exactly what the cohort in our study responded to best.

As Reynolds et al also made clear, the vast majority of e-MH research has been directed toward evaluating the impact of Web-based therapy programs. Little consideration has been given to evaluating other e-MH resources or workforce roles. Our study highlights the potential value that Indigenous and non-Indigenous health providers in support worker roles may have in using relevant mental health resources on YouTube and social media for health educational purposes, as has been successfully demonstrated in other health-related contexts [39,40]. However, the quality of such resources does need to be carefully assessed. Therefore, we suggest that much more attention be given to developing agreed criteria for evaluating apps and YouTube videos [41-43], which may play a valuable role in health service delivery in the future.

### Limitations

We recognize that this study had a number of limitations. Reliance on consultant-trainers’ reports and the interview was both a strength and a limitation: a strength because observations were recorded immediately after sessions for all 30 consultation sessions, but a limitation because these observations were “second-hand” —the consultants rather than the participants were recording the participants’ experiences. Consequently, these observations may be subject to perceptual bias on the part of the consultants. Another issue was that we did not accurately record who was using what e-MH resources other than Stay Strong prior to training and prior to the consultation sessions. We believe that only 2 of the participants had any previous interest or exposure to any form of e-MH, but this was not recorded.

Unfortunately we were unable to determine e-MH usage beyond the consultation sessions and follow up those health professionals who only attended 1 or 2 sessions, as further follow-up lay outside our ethics approval. It is therefore possible that the e-MH usage figures from the consultants’ reports may underestimate eventual usage as some participants may have started to use e-MH resources after they had ceased to attend consultation sessions.

### Conclusions

This study highlights the importance of broadening our conception and definition of e-mental health resources, which, until now, has been largely restricted to Web-based therapy programs. A growing health education literature featuring Internet-based materials, and our own study, suggest that e-mental health resources should encompass other Web-based materials (eg, apps, YouTube videos) that may promote social and emotional well-being, and can be used by a variety of different workforces that do not necessarily have a clinical role [37]. In the present case, it is suggested that for Aboriginal health providers who do not have specific health professional qualifications and are in support worker or health education roles, training in the use of health promotional e-MH resources may be rather more valuable than training in Web-based therapy programs or apps that require a coaching role. The study also highlights the importance of preparatory work with organizations that plan to send their staff to e-MH training (see the organizational checklist provided by Puszka et al [36]). Trainer-consultants need to work with these organizations to acquire necessary e-MH resources before training programs and enable their staff to attend post-training consultation sessions. This extends to such basic planning as ensuring staff have access to resources such as iPads, Androids, and iTunes accounts [36].

What we are learning is that, when it comes to translation into practice, the devil is in the detail. What is seen as valuable “on the ground” is not necessarily what is seen as valuable by university researchers. Consumer and service provider involvement is vital if e-MH is to fulfill its undoubted promise [32], particularly in disadvantaged communities such as Aboriginal and Torres Strait Islander communities [28,44].

To summarize, we suggest that the training plus consultation model was successful in enhancing engagement and usage of e-MH resources. However, a number of organizational and personal barriers to implementation also emerged. To increase efficacy, e-MH resources for Indigenous communities needs to be not only culturally relevant, but role- and organizationally relevant. In the context of Australian Indigenous health providers, role and organizational relevance means broadening the scope of e-MH resources from a focus on e-therapy to a focus on health promotion in the form of health education and information resources.

## Appendix

Multimedia Appendix 1 A deidentified example of a consultant-trainer session report (names changed).

Multimedia Appendix 2 Interview guide with the consultant-trainers.

Multimedia Appendix 3 Examples of health education and information resources explored in consultation sessions.

## Abbreviations

CT1: consultant-trainer 1
CT2: consultant-trainer 2
CTR: consultant-trainer report
e-MH: e-mental health
eMHPrac: e-Mental Health in Practice
NGO: nongovernmental organization
