# Supplementary material for: Translating E-Mental Health Into Practice: What Are the Barriers and Enablers to E-Mental Health Implementation by Aboriginal and Torres Strait Islander Health Professionals?
Source: J Med Internet Res. 2017 Jan 11;19(1):e1. doi: 10.2196/jmir.6269 (PMC5266824; doi:10.2196/jmir.6269)
Supplement: Multimedia Appendix 1 [file jmir_v19i1e1_app1.pdf]

Week 1 – Group D
Time: 1.5 hours
Present: 3 workers; 1 team leader

The organisation has bought three Ipads but they have not yet been made available for staff use or had Stay Strong downloaded on them yet. Still need to work out how to manage an iTunes account through an organisation. Staff keen for this to happen. Team Leader Jerry was present for start of booster session and said this would be a priority. Request for Stay Strong paper version, which we will give.
At present internet connection to their computer is difficult.
Hard using technology as outreach workers, but can see how Stay Strong can be used with the men's group with individual catch up.
Asked if they had referred to Mindspot. They had not nor were they aware of it. Used Ipads to look at the [Mindspot] website which interested them a lot.
Staff noticing that clients use phones a lot. Organisation uses SMS for app reminders.
Jane liked the idea of using apps to assist with medication reminders (like a Webster pack).
Trevor keen to use technology to support people with mental health issues around depression and anxiety. Had good discussion about this.
Susan identified she works a lot with children with ADHD. Explored YouTube for clips on the subject that may be helpful. Also looked at websites for young people at high school that Susan could find useful.
Questions about how Facebook could be used to assist people. Talked about organisations that have a Facebook presence that clients could be encouraged to become friends with e.g. Smiling Minds, Black Dog Institute etc.
Last part of session used to practice with Stay Strong again and set personal goals which included:

- Keep using the Stay Strong app
- Use the Stay Strong app more often
- Taking the ipad out when I meet new patients
- Getting more skilled in using the ipad

Reflection after practicing with the Stay Strong app again:

- Was good to go over the app again
- Feeling more confident with the app with more practice
- Can see how it can be used to help people with anxiety, grief and loss, e.g. helping people deal with their worries better
- Can see I will get better with the app if I practice daily just for a short time.

Staff found the eMPrac resource guide very helpful. Asked for more for other staff.
