# Supplementary material for: Translating E-Mental Health Into Practice: What Are the Barriers and Enablers to E-Mental Health Implementation by Aboriginal and Torres Strait Islander Health Professionals?
Source: J Med Internet Res. 2017 Jan 11;19(1):e1. doi: 10.2196/jmir.6269 (PMC5266824; doi:10.2196/jmir.6269)
Supplement: Multimedia Appendix 2 [file jmir_v19i1e1_app2.pdf]

1. What worked well?
    a. Can you give us some examples/stories?
    b. What were some of the positive experiences people reported in the sessions?

2. What's not worked so well?
    a. Examples/stories?
    b. What would you say were difficulties participants' reported in the sessions?

3. Based on your experiences, how would you approach the consultation sessions differently next time?
    a. At an organisational level
    b. In regards to the consultation sessions
    c. Anything else?

4. What changes did you notice in peoples' interest in new technologies over the six months?

5. What changes did you notice in peoples' use of new technologies over the six months?

6. Is there anything else that you think is important to remember for the future roll-out of consultation groups?
