# Supplementary material for: Translating E-Mental Health Into Practice: What Are the Barriers and Enablers to E-Mental Health Implementation by Aboriginal and Torres Strait Islander Health Professionals?
Source: J Med Internet Res. 2017 Jan 11;19(1):e1. doi: 10.2196/jmir.6269 (PMC5266824; doi:10.2196/jmir.6269)
Supplement: Multimedia Appendix 3 [file jmir_v19i1e1_app3.pdf]

- Examples of Youtube clips:
    - Nuggets https://www.youtube.com/watch?v=HUngLgGRJpo
    - Meth Inside Out: Brain and Behaviour. Various youtubes including: https://www.youtube.com/watch?v=dsFpe4IiF8w
    - Kids matter https://www.youtube.com/watch?v=WxH3aTVVp-o

- Stayin on track: https://www.stayinontrack.com/

- Alcohol and safety: https://itunes.apple.com/au/app/alcohol-and safety/id1017564062?mt=8

- So You Think You Can Quit app: http://nosmokes.com.au/fun/apps/

- Rays night out: https://itunes.apple.com/au/app/rays-night-out/id978589497?mt=8

- 3D Brain app: https://itunes.apple.com/au/app/3d-brain/id331399332?mt=8

- Worrytime: http://au.reachout.com/reachout-worrytime-app

- Breathe: http://au.reachout.com/reachout-worrytime-app

- My Script Smart Note: https://itunes.apple.com/us/app/myscript-smart-note-handwriting/id477967083?mt=8
